# Navigated Minimally Invasive Cervical and Cervicothoracic Fixation: A Technical Note on Surgical Technique and Proposed Classification

**DOI:** 10.7759/cureus.91414

**Published:** 2025-09-01

**Authors:** Spyridon Komaitis, Konstantinos Zygogiannis, Sotirios Karatzoglou, Dimitrios Klitsinikos, Dritan Pasku, Khalid Salem

**Affiliations:** 1 Centre for Spinal Studies and Surgery, Queen's Medical Centre, Nottingham University Hospitals, NHS Trust, Nottingham, United Kingdom; 2 Centre for Spinal Studies and Surgery, Queen's Medical Centre, Nottingham University Hospitals, NHS Trust, Nottingham, GBR

**Keywords:** cervical, cervicothoracic, fixation, minimally invasive, pedicle screw

## Abstract

The purpose of this study is to propose a standardized classification of minimally invasive cervical pedicle screw (MICEPS) fixation according to the levels instrumented and the extent of the construct, thereby facilitating reproducible surgical planning and technique.

We developed a three-tiered MICEPS classification with a specific surgical algorithm based on anatomic levels and construct length: Type 1, subaxial cervical fixation; Type 2, subaxial cervical to proximal thoracic fixation; and Type 3, subaxial cervical to T3/4 cervicothoracic stabilization. All techniques employ O-arm intraoperative navigation and preserve posterior tension-band integrity. We describe key technical steps and compare each type in terms of incision strategy, soft-tissue handling, and navigation workflow.

Each MICEPS type employs a tailored combination of small paramedian incisions, muscle-sparing dissection, and intraoperative navigation to achieve stable posterior fixation while minimizing soft-tissue trauma: Type 1 is indicated for short subaxial cervical fusions (typically C3-C6/C7). It uses a single small paramedian incision on each side of the spine and follows a purely muscle-sparing corridor. Type 2 extends the construct to T1 or T2, still via one paramedian incision per side, but involves splitting and subsequent re-approximation of the trapezius muscle. Type 3 reaches down to T3/T4, employing two mini-open incisions on each side and controlled splitting of the trapezius. All three techniques provide a safe, anatomically direct corridor without the need for significant retraction that could compromise navigation accuracy.

The provided MICEPS classification offers a clear, anatomically driven framework for minimally invasive posterior cervical and cervicothoracic fixation. By tailoring incision number, muscle-sparing corridors, and navigated instrumentation to the required fusion extent, surgeons can achieve high-precision screw placement, minimal morbidity, expedited recovery, and high repeatability. Although a formal learning curve exists, MICEPS represents a safe, cost-effective alternative to open techniques in appropriately selected patients.

## Introduction

Minimally invasive surgery (MIS) for cervical spine disorders has emerged as a significant advancement in spine surgery, propelled by innovations such as full endoscopy and advanced imaging techniques. MIS techniques, particularly those employing full-endoscopic procedures, have demonstrated clinical efficacy and improved patient satisfaction compared to traditional methods. A systematic review indicates that full endoscopic cervical surgery (FECS) frequently results in reductions in cervical and radicular pain while enhancing functional outcomes, as assessed by the visual analog scale (VAS) and the Neck Disability Index (NDI) [1]. This is corroborated by recent studies that highlight the benefits of posterior minimally invasive approaches, which minimize soft-tissue damage and contribute to quicker recovery times while preserving cervical mobility [2,3].

Minimally invasive cervical pedicle screw (MICEPS) fixation offers several advantages over traditional open techniques for posterior cervical spine fusion. One of the primary benefits is the precision achieved through the use of advanced intraoperative navigation systems such as the O-arm. The O-arm enables real-time imaging during surgery, facilitating accurate placement of screws while minimizing soft tissue disruption, which is a significant concern in open surgical techniques [4]. Studies demonstrate that MICEPS, aided by O-arm navigation, results in significantly less tissue trauma and a lower incidence of complications, such as vertebral artery injury, compared to open procedures. The precise horizontal insertion of pedicle screws is particularly effective at the midcervical spine and contributes to reducing screw perforation rates, thereby enhancing overall surgical safety [4,5].

Additionally, the MICEPS technique integrates tissue-sparing approaches that minimize muscle stripping and soft tissue retraction, which are prominent in traditional methods. This reduction in soft tissue damage correlates with decreased postoperative pain and a more rapid recovery, allowing patients to resume normal activities sooner [6]. The statistical evidence suggests that patients undergoing MICEPS experience shorter hospital stays and fewer postoperative complications, which is particularly advantageous for older patients and those with comorbid conditions. For instance, a review noted significant differences in length of stay between patients who underwent open surgery and those managed with minimally invasive techniques, underscoring the efficiency of MICEPS [7].

The approach not only enhances the functional recovery of patients but also proves beneficial in terms of economic considerations. With decreased hospital stays and reduced need for extensive rehabilitation, the cost-effectiveness of MICEPS becomes evident. Cost variations have been highlighted in literature comparing open procedures to minimally invasive techniques in terms of hospital charges and recovery costs, making MICEPS an increasingly attractive option for spine surgeons and healthcare systems aiming to optimize resources without compromising patient care [8]. As research continues to validate the benefits of MICEPS, its role is poised to expand in the field of cervical spine surgery, offering a robust alternative to conventional methods while prioritizing patient outcomes and safety.

## Technical report

Technical note and MICEPS classification

*Type 1 MICEPS* *: Subaxial Cervical Fixation Through a Single Approach on Each Side*

**Figure 1:**
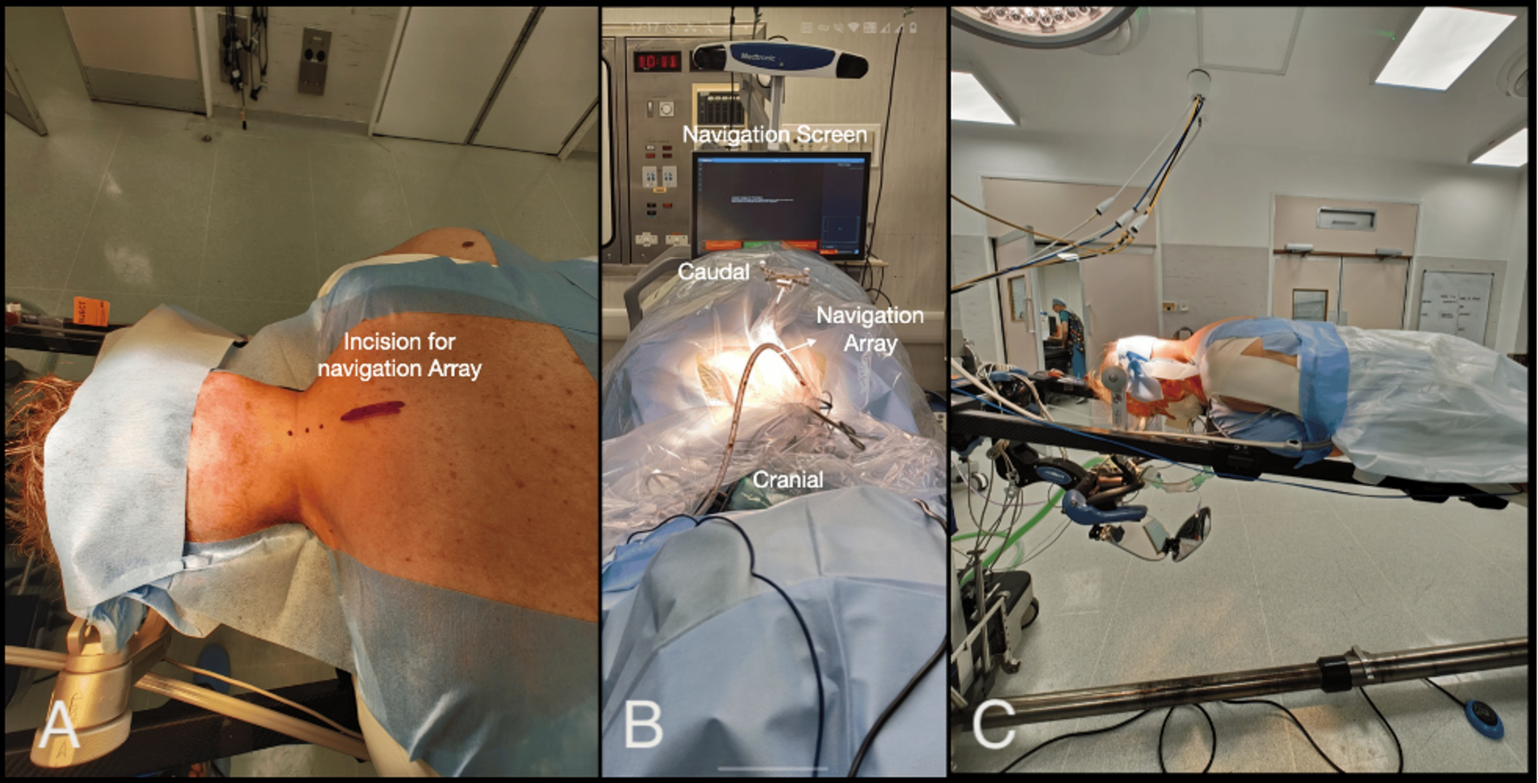
Patient positioning, array incision, and navigation screen position. (A) Left view; (B) cranial view; (C) patient positioning on the Allen table.

MICEPS fixation Type 1 is designed for posterior cervical fusion, typically spanning two to five levels, in cases of trauma, tumor, infection, or localized instability (Figure 1). This technique minimizes soft-tissue dissection and preserves posterior muscular attachments, offering the benefits of reduced blood loss, decreased postoperative pain, and faster recovery. The patient was placed in the prone position on an Allen Spinal Table. The head was secured in a Mayfield head clamp and elevated significantly compared to the lower part of the body to make the cervicothoracic junction parallel to the floor level while maintaining neutral alignment or the desired correction. The Navigation Screen was positioned adjacent to the feet of the patient. The neck and posterior cervical region were prepared and draped in the usual sterile fashion. A small midline incision was made at the level of the cervicothoracic junction, followed by attachment of the array at the spinous process of T1/T2 (Figure 1). A single spin with the O-Arm was carried out. Two small paramedian incisions were made, guided by the navigation pointer, one on each side of the midline, centered over the involved levels.

Each incision measured approximately 2.5 to 4 cm, providing access for instruments and facilitating screw placement under imaging guidance.

Blunt dissection is performed through the natural muscle planes, including the trapezius, splenius capitis, semispinalis capitis, and longissimus capitis. At this level, the orientation of the fibers is such that no sharp dissection is required. Before exposing the lateral masses of interest with a monopolar insulated tip, brisk bleeding from vascular plexuses may be encountered. This is easily controlled with packing and application of hemostatic material. A tubular or normal retractor is inserted to establish a working channel.

**Figure 2:**
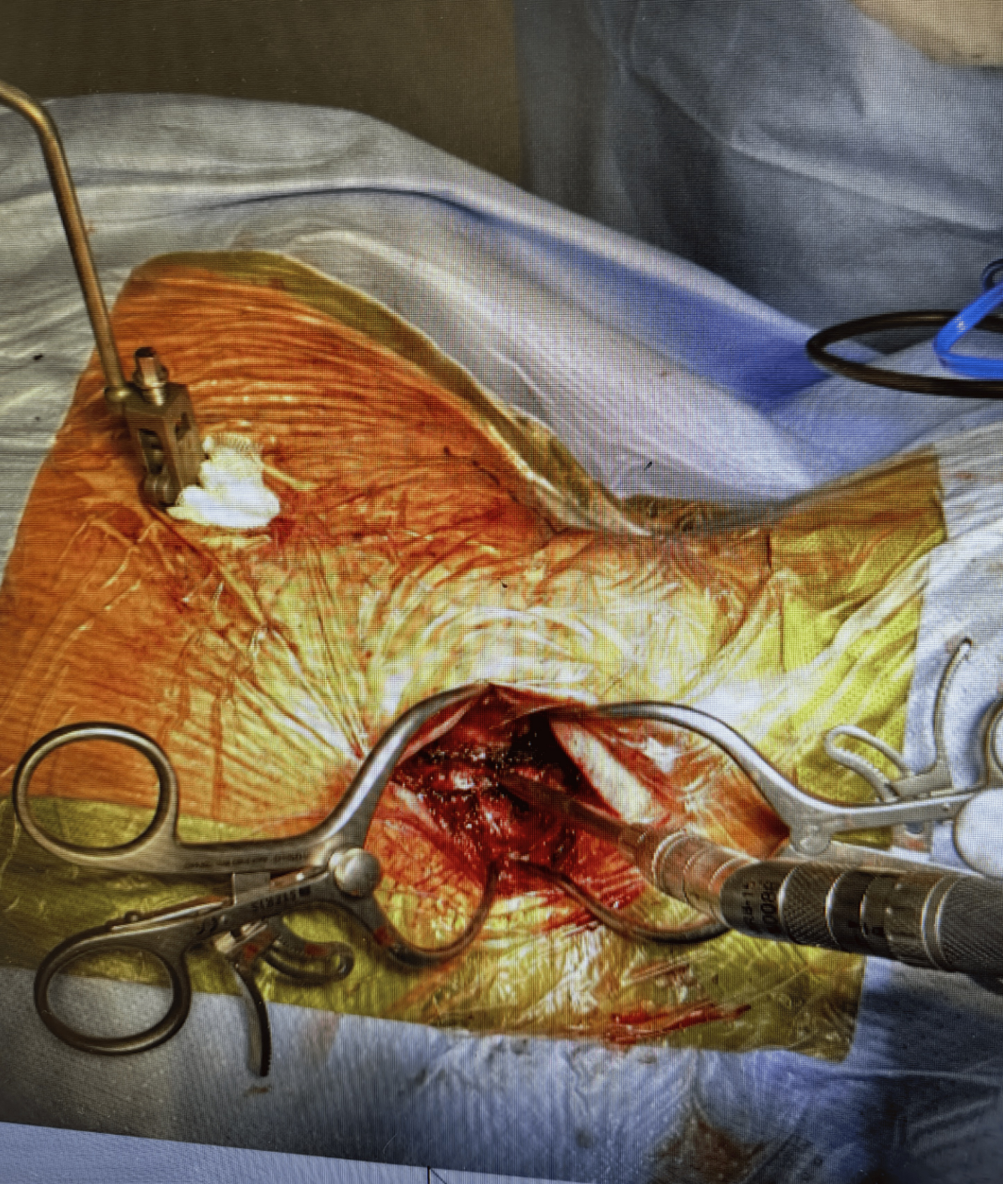
Identification of the pedicle screw entry point with a navigated burr.

**Figure 3:**
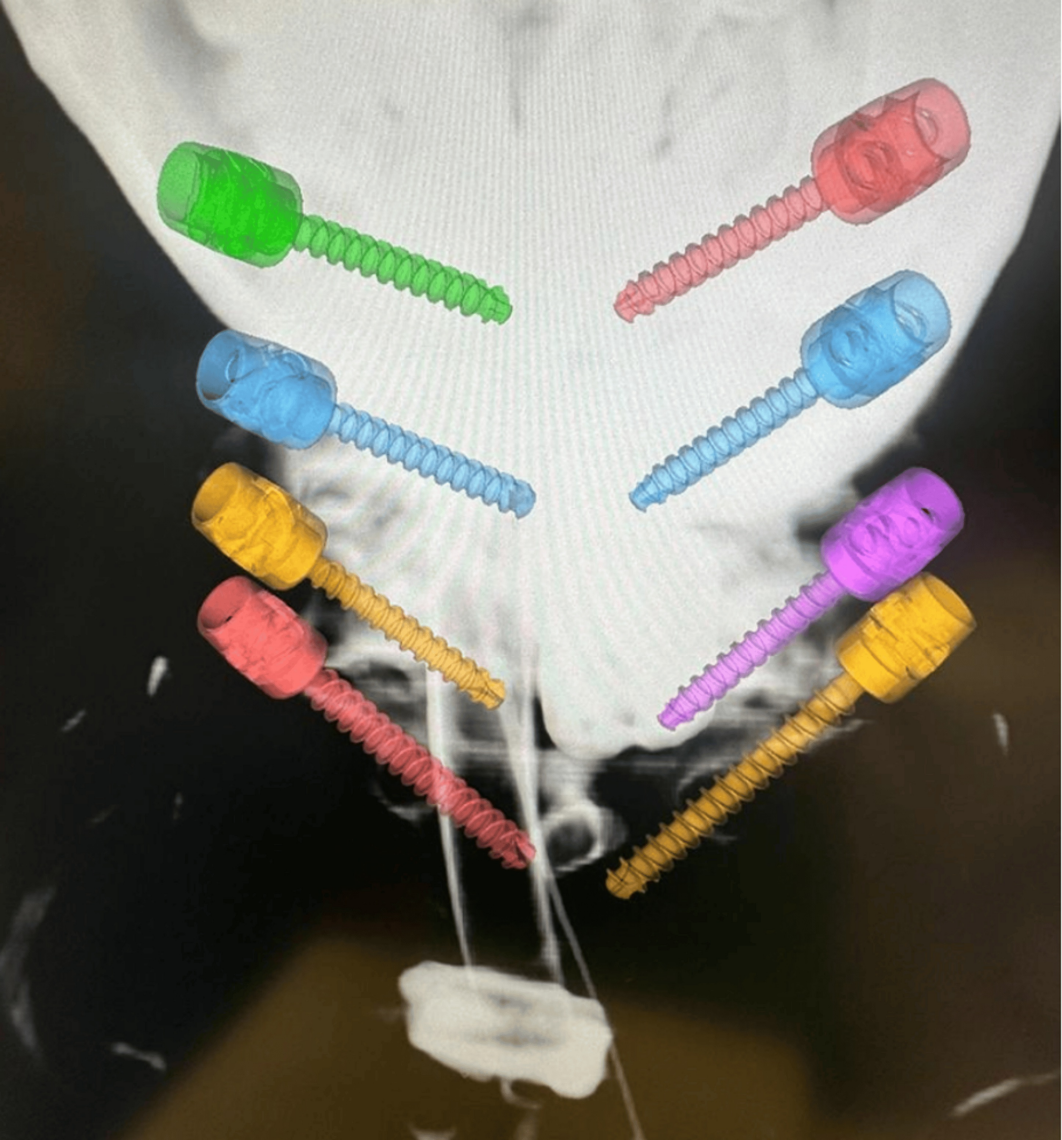
3D reconstruction of navigated cervical pedicle screws during a Type 1 MICEPS. Image credit: Spyridon Komaitis. MICEPS, minimally invasive cervical pedicle screw

Using intraoperative navigation, the pedicle entry points are identified, typically at the junction of the lateral mass and superior articular process. A navigated high-speed burr with a 2.3-matchstick burr is used to create a pilot hole at each entry point, after which the pedicle screws are carefully advanced along the planned trajectory under image guidance. It is important to note that while utilizing this approach, the trajectory of the Burr aligns with the incision with no need for retraction that might affect navigation accuracy (Figure 2). The use of triggered electromyographic monitoring is recommended to minimize the risk of nerve root injury. Screw diameters generally range from 3.5 to 4.0 mm, depending on patient anatomy (Figure 3).

**Figure 4:**
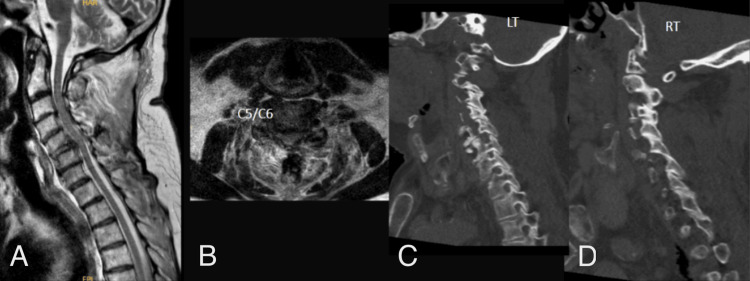
C5-C6 fracture with associated disc injury and prevertebral hematoma, selected for Type 1 MICEPS following ACDF. (A) Mid-sagittal T2 MRI; (B) axial T2 MRI view of the index level; (C) paramedian left view, CT scan; (D) paramedian right view, CT scan. MICEPS, minimally invasive cervical pedicle screw; ACDF, anterior cervical discectomy and fusion

**Figure 5:**
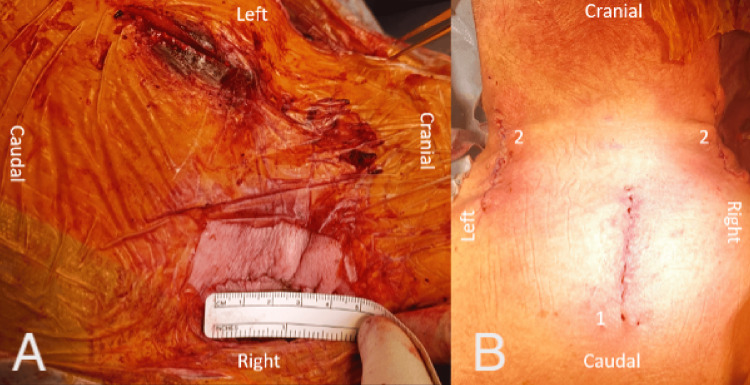
Skin incisions used for Type 1 MICEPS in the case illustrated in Figure 4. (A) Top right view; (B) top midline view. 1: Midline incision for attachment of navigation array; 2: bilateral incisions for screw placement. MICEPS, minimally invasive cervical pedicle screw

**Figure 6:**
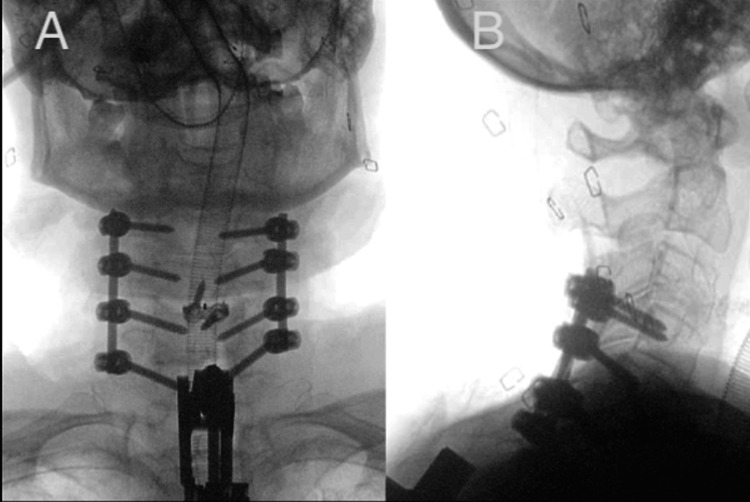
X-rays of Type 1 MICEPS extending from C4 to T7 for the case illustrated in Figure 4. (A) AP X-ray; (B) lateral X-ray. MICEPS, minimally invasive cervical pedicle screw; AP, anteroposterior

Once pedicle screws are placed bilaterally, pre-contoured rods are introduced through the mini incisions. Rod holders or specialized rod inserters designed for minimally invasive applications guide the rods into position within the screw heads. The construct is assembled, and final tightening is performed sequentially, ensuring preservation of alignment and stability. After confirming hardware placement, meticulous hemostasis is achieved, and the retractors are removed, allowing the musculature to fall back into its natural position. The fascia, subcutaneous tissues, and skin are closed in layers using absorbable sutures and skin adhesive or subcuticular suturing techniques. This technique emphasizes the importance of precise screw trajectory planning, facilitated by intraoperative navigation, given the limited visualization inherent to minimal access approaches. Type 1 MICEPS is particularly suited for short constructs, offering stable fixation with minimal disruption of the posterior soft-tissue envelope. Surgeons employing this technique should have expertise in cervical pedicle screw placement and minimally invasive spinal instrumentation (Figures 4-6).

*Type 2 MICEPS* *: Subaxial Cervical to Proximal Thoracic Fixation With One Incision on Each Side*

Type 2 MICEPS is indicated for short cervicothoracic posterior fusion (up to 4-5 levels) in conditions such as trauma, tumor, infection, or focal instability involving the lower cervical spine and upper thoracic spine (Figure 4). This technique is typically applied to constructs spanning C5-T1, C6-T2, or similar levels where the cervicothoracic junction requires rigid stabilization while minimizing soft-tissue disruption. Type 2 MICEPS is ideal for cases where preservation of paraspinal musculature and reduction of surgical morbidity are desired.

**Figure 7:**
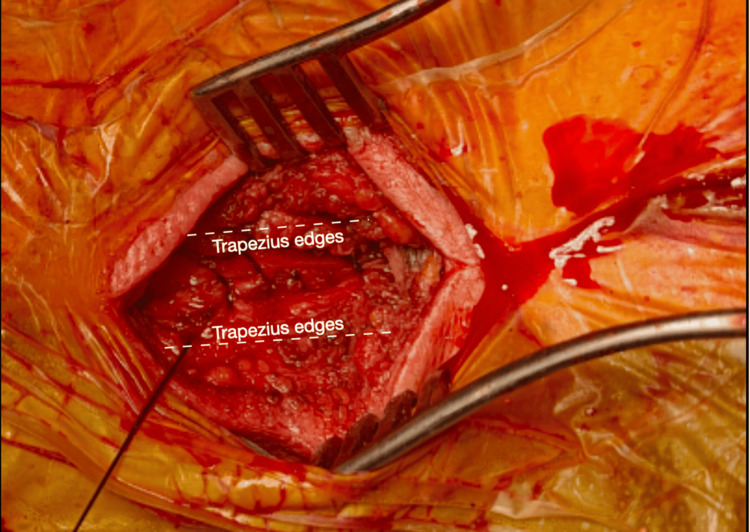
Reapproximation of the trapezius edges using a continuous suture during closure.

**Figure 8:**
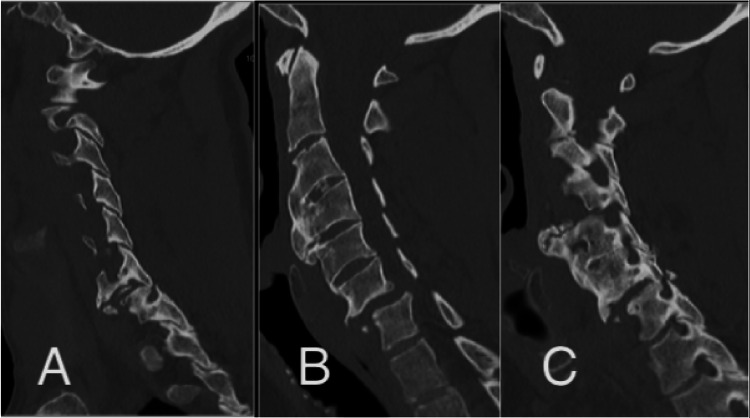
Preoperative CT scan demonstrating an unstable fracture-dislocation at the C6-C7 level, treated with Type 2 MICEPS following ACDF and plating. (A) Left paramedian view; (B) mid-sagittal view; (C) right paramedian view. MICEPS, minimally invasive cervical pedicle screw; ACDF, anterior cervical discectomy and fusion

**Figure 9:**
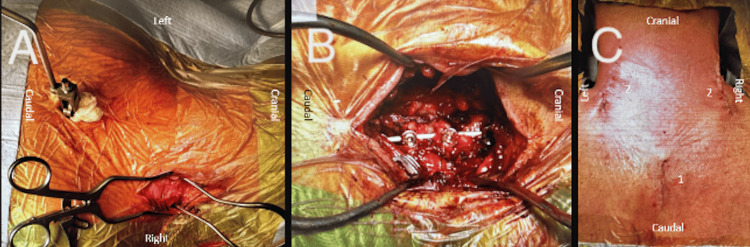
Intraoperative demonstration of Type 2 MICEPS used in the treatment of the illustrated case. (A) Right-sided view; (B) right-sided incision close-up view; (C) midline view. 1: Midline incision for attachment of navigation array; 2: bilateral incisions for screw placement. MICEPS, minimally invasive cervical pedicle screw

**Figure 10:**
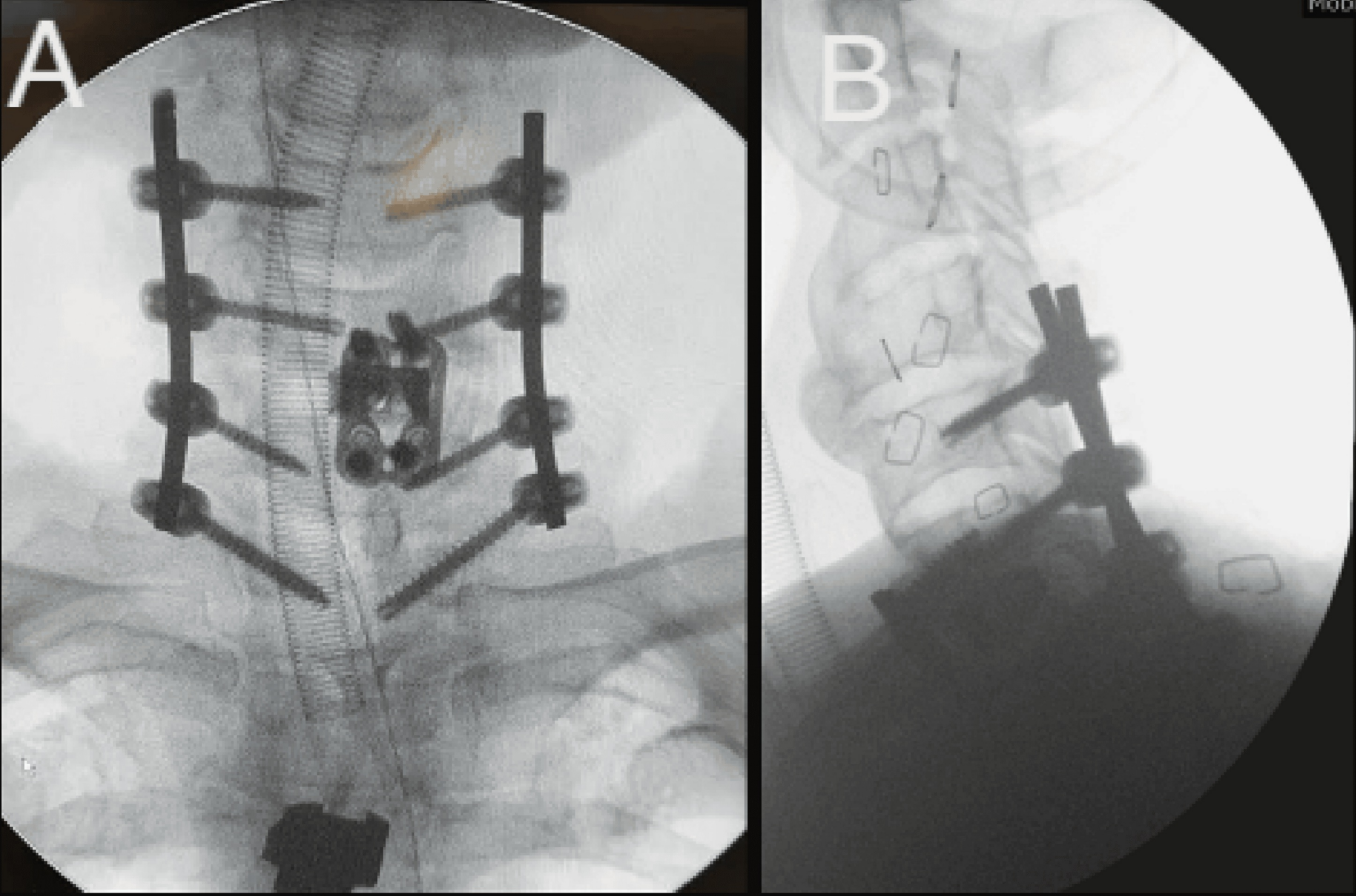
X-rays of Type 2 MICEPS spanning C5 to T1 used for treatment of the illustrated case. (A) AP X-ray; (B) lateral X-ray. MICEPS, minimally invasive cervical pedicle screw

The technique utilizes one small paramedian incision on each side, positioned to allow access to both the lower cervical and upper thoracic pedicles. Dissection through muscle planes is performed. The main difference with the Type 1 MICEPS is that at this level, the fibers of the Trapezius follow a direction perpendicular to the orientation of the dissection. For this reason, the trapezius is carefully dissected using monopolar diathermy, utilizing tuck-up sutures for reapproximation during closure (Figure 7). The muscles deep to the trapezius are then dissected bluntly along the trajectory of their fibers. Tubular or expandable retractors provide a working corridor. Pedicle screws are placed into the cervical and upper thoracic levels under navigation. Pre-contoured rods are introduced through the mini incisions and secured to the screws with minimal soft-tissue manipulation. This construct provides stable fixation across the cervicothoracic junction while preserving the integrity of the posterior musculature (Figures 7-10).

*Type 3 MICEPS*​​​​​​*: Subaxial Cervical to T3/T4 Fixation With Two Mini Incisions on Each Side*

**Figure 11:**
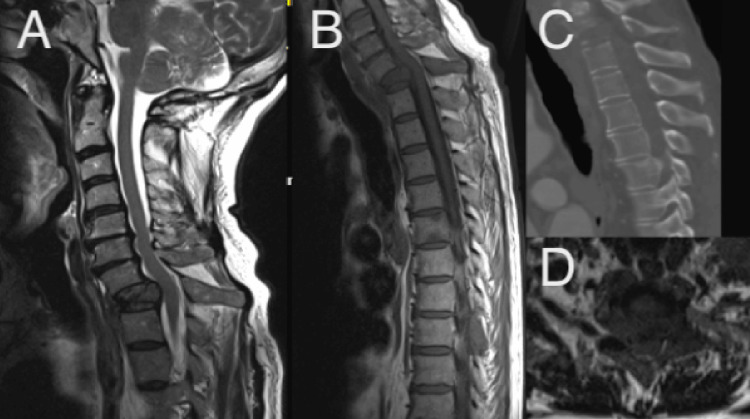
Metastatic disease at T1 with concomitant instability, treated with anterior corpectomy followed by Type 3 MICEPS. (A) MRI T2 sagittal plane; (B) MRI T1 sagittal plane; (C) CT sagittal plane; (D) MRI T2 axial view at the index level. MICEPS, minimally invasive cervical pedicle screw; MRI, magnetic resonance imaging

Type 3 MICEPS is intended for long-segment posterior stabilization of the cervicothoracic spine in complex cases where extensive fixation is required. Typical indications include unstable fractures through ankylosed spines (such as in ankylosing spondylitis or diffuse idiopathic skeletal hyperostosis), pathological fractures from neoplasms, multilevel infection, and severe deformity or instability involving both the cervical and upper thoracic spine. Constructs generally span from mid-cervical levels down to T3, T4, or even lower, depending on the extent of the pathology and the need for biomechanical support across the cervicothoracic junction (Figure 11).

**Figure 12:**
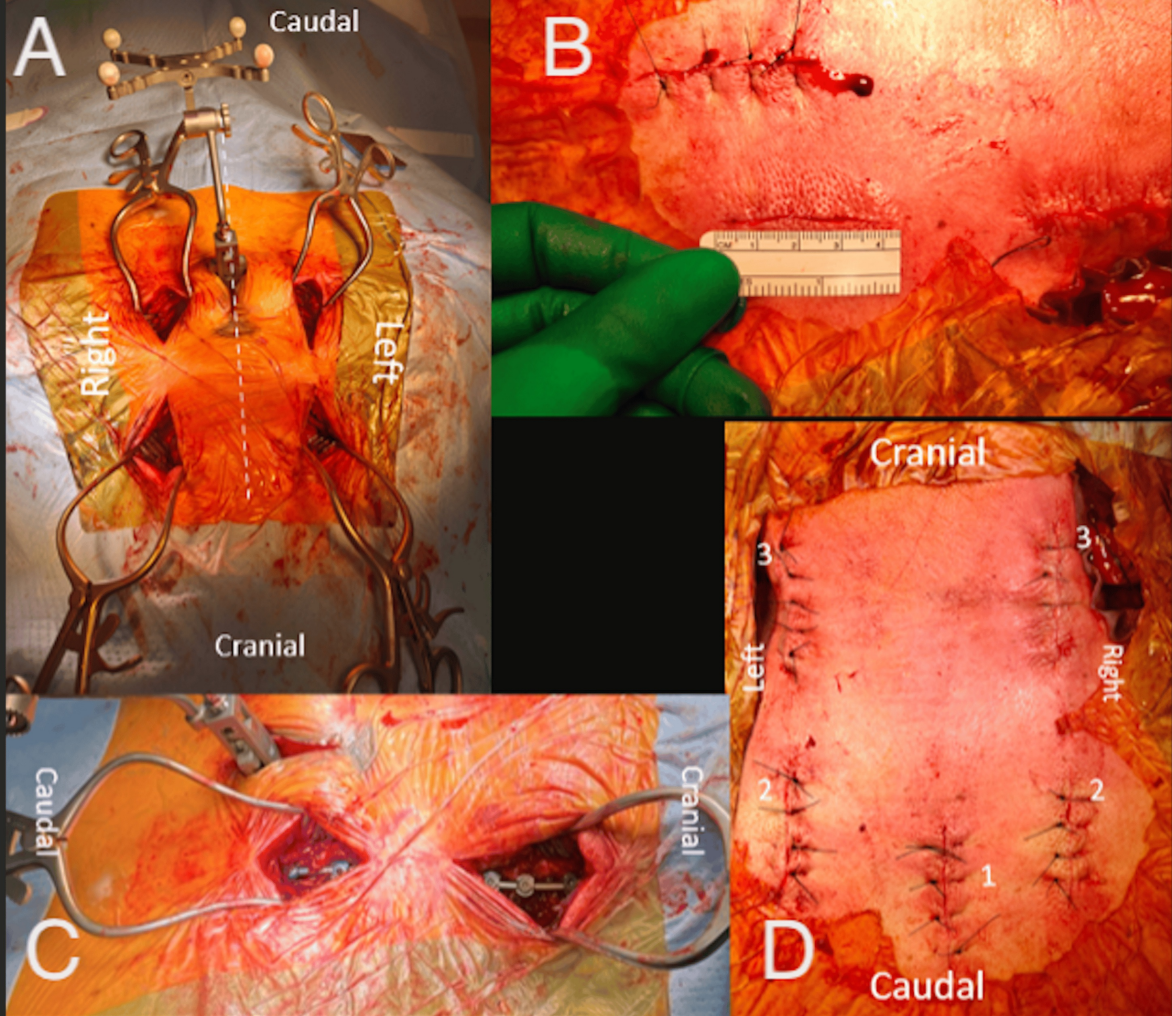
Intraoperative demonstration of Type 3 MICEPS as utilized for the illustrated case. (A) Top view - midline intraoperative; (B) right view - demonstration of incision length; (C) top-right view; (D) top view - midline after closure of incisions. 1: Midline incision for attachment of navigation array; 2: bilateral incisions for thoracic screw placement; 3: bilateral incisions for subaxial cervical screw placement. MICEPS, minimally invasive cervical pedicle screw

**Figure 13:**
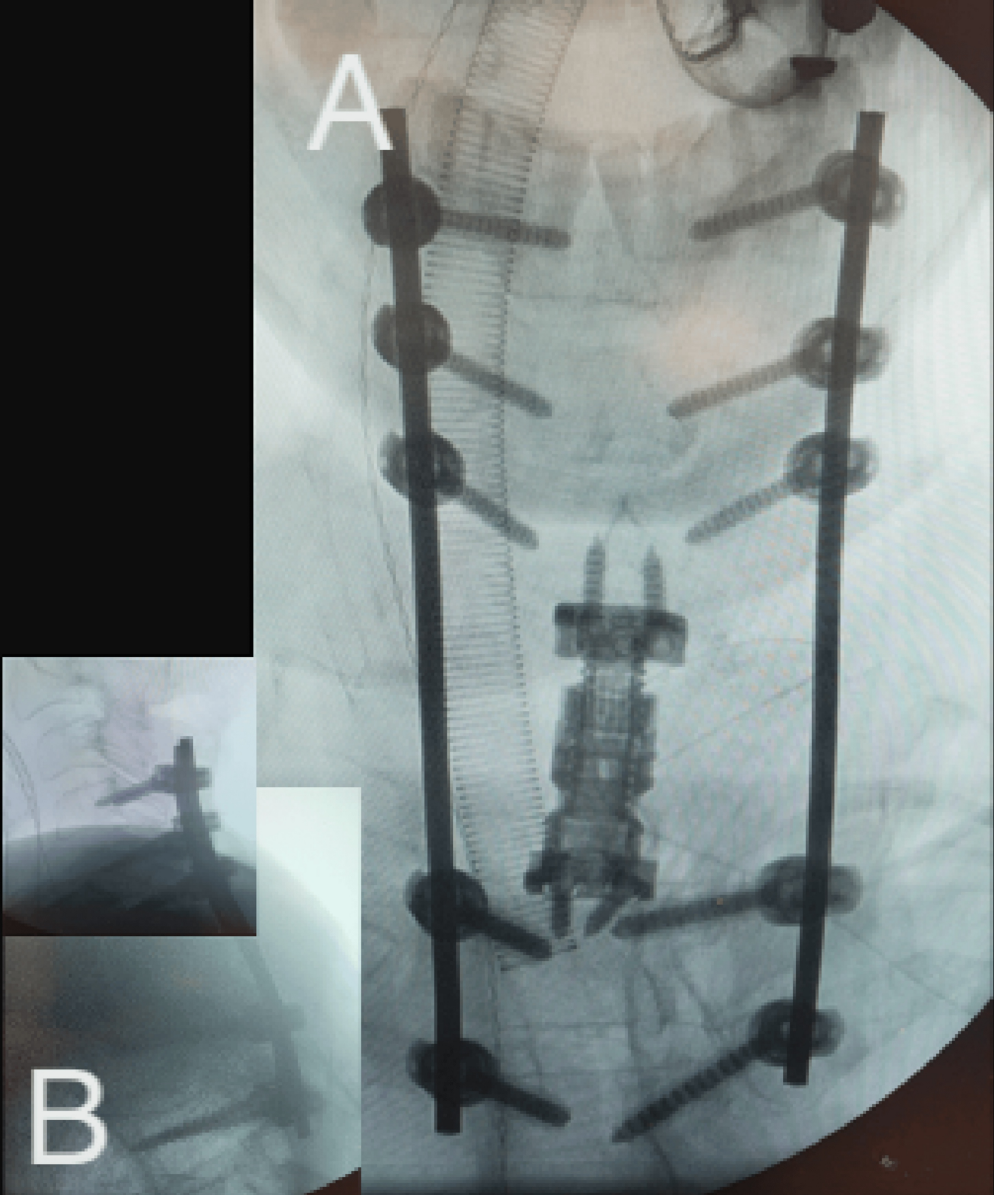
X-rays of Type 3 MICEPS spanning C4 to T3, as utilized for the illustrated case. (A) AP X-ray; (B) lateral X-ray. MICEPS, minimally invasive cervical pedicle screw

As noted before, a small midline incision is utilized at the level of the intended lower instrumented level. The navigation array is attached, and a spin is carried out with the C-arm. For long fixations, a second spin might be required to include all required levels. The surgical approach involves two small paramedian incisions on each side of the posterior neck and upper back guided by the navigation probe. These incisions are strategically positioned to provide access to the target cervical and thoracic pedicles along the length of the construct. For the cranial incisions, blunt dissection is used to create working channels through the muscle planes, similar to the technique described in Type 1 MICEPS, and tubular or normal retractors are deployed to maintain exposure. For the caudal incisions, the approach is similar to that described in Type 2 MICEPS above. In this area, the trapezius needs to be dissected with the monopolar and reapproximated with sutures at the end of the procedure. Pedicle screw placement at these levels poses unique challenges due to the narrow pedicles in the cervical spine, the transition in anatomy at the cervicothoracic junction, and the frequent presence of altered or fused anatomy in cases of ankylosing disorders. The procedure is identical to that described above, using a navigated burr with a 2.3-mm matchstick to create the pilot holes, followed by screw insertion. Intraoperative navigation is essential for precise trajectory planning, and neuromonitoring (e.g., triggered electromyography (EMG)) is useful for reducing the risk of neural injury. The placement of pre-contoured rods through multiple small incisions and their secure engagement into the screw heads can be technically demanding, requiring careful rod contouring and sequential assembly to avoid soft-tissue entrapment or misalignment. Our technique involves the insertion of a rod contoured according to the individual alignment of the cervicothoracic junction, which is inserted through the caudal incision and advanced from caudal to cranial. Type 3 MICEPS allows for rigid, long-segment fixation with minimal disruption of posterior soft tissues but requires advanced surgical skill and experience with both cervical pedicle instrumentation and minimally invasive spinal techniques (Figures 12-13). All types of MICEPS approaches and related techniques are summarized in Table 1.

**Table 1:** Summary of MICEPS types and associated technical aspects. MICEPS, minimally invasive cervical pedicle screw

Type	Extent	Number of paramedian incisions	Dissection of muscles	Insertion of the rod	Position of the array
Type 1 MICEPS	Subaxial cervical spine (C3-C7)	One incision on each side	Blunt dissection alongside the direction of muscles	Under direct vision	Spinous process of the distal instrumented vertebra or the Mayfield Clamp
Type 2 MICEPS	Subaxial cervical spine to proximal thoracic spine (T1/T2)	One incision on each side	Splitting of the Trapezius is required, followed by suturing during closure.	Under direct vision	Spinous process of the distal instrumented vertebra or the Mayfield Clamp
Type 3 MICEPS	Subaxial Cervical Spine to T3/T4	Two incisions on each side.	Splitting of the Trapezius is required, followed by suturing during closure.	Inserted through a caudal incision and advanced cranially below the fascia	Spinous process of the distal instrumented vertebra or the Mayfield Clamp

## Discussion

The comparison between MICEPS techniques and traditional open posterior cervical spine fusion highlights significant differences in complication rates, operative metrics, and postoperative recovery, supporting the growing preference for minimally invasive strategies in appropriate cases. A growing body of literature consistently reports that MICEPS techniques are associated with markedly lower major complication rates. While traditional open posterior cervical fusion has been linked with complication rates of up to 30% in various series, largely due to greater soft-tissue disruption, higher blood loss, and longer operative times [9], MICEPS procedures demonstrate major complication rates generally below 10%, reflecting the protective effect of minimized dissection and less traumatic exposure [9]. These complications in open surgery include wound dehiscence, deep infections, significant blood loss requiring transfusion, and neurovascular injuries, all of which contribute to prolonged recovery and increased healthcare costs.

Operative duration is another critical parameter distinguishing MICEPS from traditional open techniques. Published data indicate that MICEPS can typically be performed in an average of 80-100 minutes, depending on the number of levels and the complexity of the pathology, whereas traditional open posterior cervical fusion for similar indications may extend to 150-200 minutes [10,11]. The reduction in operative time not only decreases anesthetic risk but is also associated with lower intraoperative blood loss, which is significantly reduced in MICEPS, with average losses reported at 100-150 mL compared to 400-600 mL in open procedures [11,12]. This has direct implications for the need for transfusion and associated perioperative morbidity.

Technological advances have further enhanced the safety profile and precision of MICEPS. The integration of intraoperative navigation systems, particularly O-arm-based three-dimensional imaging, has been pivotal in improving pedicle screw placement accuracy, which is critical in the cervical spine, given the narrow pedicle corridors and proximity to neurovascular structures. Studies have demonstrated that O-arm navigation achieves screw placement accuracy rates exceeding 95%, which is significantly higher than the 80%-85% accuracy reported with conventional C-arm fluoroscopy [13]. This increased accuracy reduces the risk of neurovascular complications, malpositioned hardware, and the need for revision surgery. Furthermore, the novel C-arm-free navigation techniques, as described by Zygogiannis et al. [14], illustrate how the elimination of intraoperative C-arm use can significantly reduce cumulative radiation exposure for both patients and the surgical team without compromising the precision of instrumentation. Such innovations align with modern principles of reducing occupational hazards in spine surgery.

Postoperative recovery metrics further favor MICEPS. The minimally invasive nature of the procedure translates into faster mobilization and shorter hospitalization. Patients undergoing MICEPS are typically discharged within one to two days postoperatively, while those managed with traditional open posterior cervical fusion often require inpatient care for five to seven days, or longer in cases complicated by wound issues or systemic complications [10,11]. Reduced hospital stay not only benefits patient satisfaction and recovery but also has substantial economic implications. Prolonged hospitalization and postoperative rehabilitation, often necessitated by open surgical complications, can increase total healthcare costs by more than twofold compared to minimally invasive approaches [9]. Additionally, early mobilization reduces the risk of hospital-acquired conditions such as pneumonia and venous thromboembolism, further contributing to the safety profile of MICEPS.

Across all three MICEPS subtypes, the use of the O-arm for real-time, three-dimensional imaging can facilitate consistently accurate screw trajectories and markedly reduced rates of pedicle breach, particularly in anatomically challenging mid-cervical and cervicothoracic segments. By confining dissection to natural muscle planes and limiting bony exposure through 2.5-4 cm paramedian incisions, MICEPS not only preserves the integrity of the posterior tension band, thereby minimizing the risk for adjacent segment disease, but also reduces blood loss and postoperative pain.

In our experience, Type 1 MICEPS constructs (C3-C7 ) allow focused stabilization of isolated subaxial pathologies with negligible muscle injury and rapid functional recovery. Type 2, extending from C5 to T2, likewise achieves robust cervicothoracic support without the extensive muscle stripping required in open approaches, while Type 3, providing long-segment fixation down to T3/T4, offers a feasible and less invasive alternative to open techniques, even in extensive pathologies such as ankylosed or neoplastic spines that pose significant intraoperative risks. The integration of standardized patterns of incision and approach through a paramedian corridor, intraoperative navigation guidance to safely approach the area of interest, and navigated high-speed burrs to prepare for screw insertion facilitates the reproducibility and safety of these techniques.

It is important to acknowledge that while MICEPS offers clear advantages, the technique is not without challenges. The learning curve associated with cervical pedicle screw placement via minimally invasive techniques is significant, requiring mastery of advanced navigation systems, precise anatomical knowledge, and expertise in rod delivery through minimal access corridors. Moreover, certain pathologies, such as extensive deformities with severe rotational or coronal imbalance, may still necessitate traditional open approaches for adequate exposure and correction. Nevertheless, the current evidence base strongly supports the role of MICEPS as an effective, safe, and resource-efficient alternative to open posterior cervical fusion in selected patients, particularly when augmented by modern navigation technologies.

## Conclusions

MICEPS techniques have transformed the approach to posterior cervical spine fusion by significantly reducing tissue disruption compared to traditional open surgery. This less invasive method leads to lower complication rates, including reduced risk of infection and neurological injury. Patients benefit from shorter operative times and experience less blood loss during surgery, which contributes to a quicker overall recovery. The minimally invasive approach also allows for smaller incisions, resulting in decreased postoperative pain and improved cosmetic outcomes. Advances in imaging guidance have enhanced the precision of screw placement, which is critical in the anatomically complex cervical spine. Additionally, ongoing developments in technology and surgical technique continue to optimize safety while addressing concerns such as radiation exposure during fluoroscopic guidance. With faster hospital discharge times and quicker return to daily activities, these techniques support better patient satisfaction and functional outcomes. Overall, MICEPS represent a significant advancement in spine surgery, offering a safer and more efficient alternative that aligns well with modern goals of patient-centered care. As surgical expertise and technology continue to improve, minimally invasive methods are likely to become the standard of care in cervical spine stabilization.

## References

[1] Liawrungrueang W, Cho ST, Sharma A (2025). Clinical outcomes and patient perspectives in full endoscopic cervical surgery: a systematic review. Neurospine.

[2] Lv J, Mei J, Feng X, Tian X, Sun L (2022). Clinical efficacy and safety of posterior minimally invasive surgery in cervical spondylosis: a systematic review. J Orthop Surg Res.

[3] Hur JW, Lee S, Kim B, Kim S (2023). The growing popularity of MISS: a focus on endoscopic surgery for the cervical and thoracic spine. J Minim Invasive Spine Surg Tech.

[4] Sugimoto Y, Hayashi T, Tokioka T (2017). Minimally invasive cervical pedicle screw fixation via the posterolateral approach for metastatic cervical spinal tumors. Spine Surg Relat Res.

[5] Lewis CS, Stone LE, Forseth KJ, Pham MH (2023). Minimally invasive C1-3 posterior spinal fusion with intraoperative O-arm navigation: 2-dimensional operative video. Oper Neurosurg.

[6] Lorio MP, Nunley PD, Heller JE, McCormack BM, Lewandrowski KU, Block JE (2024). Clinical implementation of tissue-sparing posterior cervical fusion: addressing market access challenges. J Pers Med.

[7] Tanenbaum JE, Lubelski D, Rosenbaum BP, Benzel EC, Mroz TE (2017). Propensity-matched analysis of outcomes and hospital charges for anterior versus posterior cervical fusion for cervical spondylotic myelopathy. Clin Spine Surg.

[8] Asthana S, Bajaj P, Staub J (2025). Comparison of RVU reimbursement in anterior or posterior approach for single- and multilevel cervical spinal fusion. Clin Spine Surg.

[9] Tanaka M, Zygogiannnis K, Sake N (2023). A C-arm-free minimally invasive technique for spinal surgery: cervical and thoracic spine. Medicina (Kaunas).

[10] Passias PG, Marascalchi BJ, Boniello AJ (2017). Cervical spondylotic myelopathy: national trends in the treatment and peri-operative outcomes over 10years. J Clin Neurosci.

[11] Agarwal N, Heary RF, Agarwal P (2018). Adjacent-segment disease after thoracic pedicle screw fixation. J Neurosurg Spine.

[12] Porter M, Schmitz MA (2022). ACDF and posterior spinal fusion revision for posterior nonunion with deformity, myelopathy, and osteoporosis in an 87-year-old: a case report and literature review. Int J Surg Case Rep.

[13] Dhillon CS, Jakkan MS, Dwivedi R, Medagam NR, Jindal P, Ega S (2018). Outcomes of unstable subaxial cervical spine fractures managed by posteroanterior stabilization and fusion. Asian Spine J.

[14] Zygogiannis K, Tanaka M, Sake N (2023). Our C-arm-free minimally invasive technique for spinal surgery: the thoracolumbar and lumbar spine-based on our experiences. Medicina (Kaunas).

